# Relations as patterns: bridging the gap between OBO and OWL

**DOI:** 10.1186/1471-2105-11-441

**Published:** 2010-08-31

**Authors:** Robert Hoehndorf, Anika Oellrich, Michel Dumontier, Janet Kelso, Dietrich Rebholz-Schuhmann, Heinrich Herre

**Affiliations:** 1 European Bioinformatics Institute, Wellcome Trust Genome Campus, Hinxton, Cambridge CB10 1SD, UK; 2 Institute for Medical Informatics, Statistics and Epidemiology, University of Leipzig, Haertelstrasse 16-18, Leipzig 04107, Germany; 3 Department of Evolutionary Genetics, Max Planck Institute for Evolutionary Anthropology, Deutscher Platz 6, Leipzig 04103, Germany; 4 Department of Biology, Institute of Biochemistry and School of Computer Science, Carleton University, 1125 Colonel By Drive, Ottawa, Ontario K1 S 5B6, Canada; 5 Department of Genetics, University of Cambridge, Downing Street, Cambridge CB2 3EH, UK

## Abstract

**Background:**

Most biomedical ontologies are represented in the OBO Flatfile Format, which is an easy-to-use graph-based ontology language. The semantics of the OBO Flatfile Format 1.2 enforces a strict predetermined interpretation of relationship statements between classes. It does not allow flexible specifications that provide better approximations of the intuitive understanding of the considered relations. If relations cannot be accurately expressed then ontologies built upon them may contain false assertions and hence lead to false inferences. Ontologies in the OBO Foundry must formalize the semantics of relations according to the OBO Relationship Ontology (RO). Therefore, being able to accurately express the intended meaning of relations is of crucial importance. Since the Web Ontology Language (OWL) is an expressive language with a formal semantics, it is suitable to de ne the meaning of relations accurately.

**Results:**

We developed a method to provide definition patterns for relations between classes using OWL and describe a novel implementation of the RO based on this method. We implemented our extension in software that converts ontologies in the OBO Flatfile Format to OWL, and also provide a prototype to extract relational patterns from OWL ontologies using automated reasoning. The conversion software is freely available at http://bioonto.de/obo2owl, and can be accessed via a web interface.

**Conclusions:**

Explicitly defining relations permits their use in reasoning software and leads to a more flexible and powerful way of representing biomedical ontologies. Using the extended langua0067e and semantics avoids several mistakes commonly made in formalizing biomedical ontologies, and can be used to automatically detect inconsistencies. The use of our method enables the use of graph-based ontologies in OWL, and makes complex OWL ontologies accessible in a graph-based form. Thereby, our method provides the means to gradually move the representation of biomedical ontologies into formal knowledge representation languages that incorporates an explicit semantics. Our method facilitates the use of OWL-based software in the back-end while ontology curators may continue to develop ontologies with an OBO-style front-end.

## Background

The OBO Flatfile Format [1] is used to represent most biomedical ontologies, among them the Gene Ontology (GO) [2] and most of the OBO Foundry ontologies [3]. To achieve interoperability between ontologies of the life sciences and semantic web ontologies, a formal semantics for the OBO format is important. While several mappings from OBO to OWL exist [1,4-6], none provides a flexible representation of the OBO semantics that corresponds with the intended meaning of the ontology developers. The OBO Relationship Ontology (RO) [7] has been adopted as the reference resource for the semantics of relations within the OBO Foundry. The current mappings between OBO and OWL do not provide the means to take the RO into consideration. The RO provides definitions for relations used in the OBO ontologies in first-order logic, of which the logic implemented by OWL is a fragment. However, the current mappings of OBO to OWL do not respect the specificity of the considered relation because a relation *R *between the categories *C *and *D *is uniformly translated as C subClassOf: R some D (using the Manchester OWL Syntax [8]).

A mapping of an OBO ontology to OWL that ignores the meaning of the relations fails to comply with OBO Foundry criteria and leads to incorrect representations. For example, the problem arises with the **lacks-part **relation, which is used in some biomedical ontologies, although not included in the RO. The meaning of *C ***lacks-part ***D *is that all instances of *C *have *no *instance of *D *as part (C subClassOf: not (has-part some D)) [9,10], yet current mappings translate it to C subClassOf: lacks-part some D. The latter statement implies the presence of an instance of *D *where the **lacks-part **relation conveys that such an instance does not exist.

Our proposal aims to contribute to the further development of the syntax and semantics of the OBO Flatfile Format, based on the assumption that any OWL axiom with two variables for classes defines a relation between these classes. Additionally, we aim to provide a method for implementing and further developing the RO and related ontologies in such a way that the relations and their definitions become amenable for automated reasoning using OWL. Furthermore, our method can be used to provide an easy-to-use OBO-style interface to complex OWL ontologies by inferring relations between OWL classes using automated reasoning. Combining the steps of our method enables the use of automated reasoning and other Semantic Web technologies for existing biomedical ontologies. It also contributes to making complex OWL ontologies available to domain experts in an OBO-style graph representation.

These are important steps towards bridging the gap between biomedical ontologies and Semantic Web ontologies. It allows for the reuse of the myriad of Semantic Web tools, methods and libraries in the domain of biomedical ontologies, and paves the way for the gradual move towards using powerful knowledge representation languages such as OWL to represent, process and query biomedical domain ontologies.

## Methods

### OBO Relationship Ontology

The semantics of relations used in biomedical ontologies is provided by the OBO Relationship Ontology (RO) [7]. The RO defines relations between *classes *using relations between instances of these classes. For example, the definition of the **part-of **relation is:

(1) $$Cpart-ofC_{1}{=}_{def}\text{for all }c,t\text{, if }Cct\text{ then there is some }c_{1}\text{ such that }C_{\text{1}}c_{\text{1}}t\text{ and }c\;part\text{-}ofc_{\text{1}}\;att\text{.}$$

This definition states that the relation **part-of **- a relation between classes (of continuants) - holds between *C *and *C*_1_, when for every instance of *C *some instance of *C*_1 _exists such that these stand in the relation *part-of *- a relation between individuals. Continuants are instantiated or have parts only at specific time points. Therefore, the universally quantified temporal argument *t *is *used*, in the definition.

In the definition of a relation between two classes *C *and *D*, these symbols are interpreted as variables varying over classes. Whenever this relation is *used*, such as in the statement

(2) Nucleus part-of Cell

the variables are replaced with the actual class names.

The RO distinguishes two categories, *Occurrents *and *Continuants*. Most relations for continuants, including instantiation or parthood, are ternary and include a temporal argument, i.e., *at which time *the continuant instantiates a class or is part of another continuant. In the definitions for binary relations between classes provided by the RO, the temporal argument in relations between individuals is universally quantified. OWL only supports binary relations between individuals, and consequently, no explicit definition of the RO definition patterns is possible in OWL.

Currently, the RO provides only natural language definitions and formal definitions in first order logic [11] for relations between classes. The OWL implementation of the RO consists of a list of OWL Object Properties [12], but does not include the definition patterns, i.e., *how *a relation between classes is reformulated using the OWL Object Properties.

### Relational pattern definitions

While the RO defines relations using first order logic, we are interested in using OWL as a knowledge representation language for biomedical ontologies. OWL is based on description logics and, in contrast to full first order logic, decision procedures are available to support automated reasoning about OWL ontologies.

We introduce a new type in OWL which represents a relation specification. To provide compatibility with the OBO Flatfile Format [1], we focus on binary relations between classes first and extend our method later. Any OWL class axiom in which two variables for classes occur represents a binary *relational pattern definition*. When a binary pattern is *applied *to two OWL class descriptions, the variable symbols are replaced with the class descriptions to yield a valid OWL class axiom. Therefore, *relational pattern definitions *are templates for complex OWL axioms.

### Extensions to OWL Syntax and Semantics

To permit human agents to understand and specify relational pattern definitions, we have extended the Manchester OWL Syntax [8] with the variable symbols ?X and ?Y. Both are symbols that are intended to represent *classes*.

This extension introduces variables for classes in OWL, and in order to remain within a decidable fragment of first order logics, we do not use a higher-order semantics for statements involving these class-variables. Although it is possible to give a decidable semantics for relations between classes in OWL [13], we do not need to use relations that contain class variables. In the OBO Flatfile Format, the relations are always used between named classes. Therefore, the variables in the extended OWL syntax are always filled by a named class. Consequently, every *application *of a relational pattern definition translates to a valid OWL axiom.

For example, we can provide a definition for the pattern **lacks-part **using the following definition:

?X subClassOf (not has-part ?Y)

Whenever this pattern is applied, it is asserted to hold between two named classes, *C *and *D*. In the semantics, this definition together with an assertion that *C ***lacks-part ***D *is expanded to the OWL statement

C subClassOf (not has-part D)

More complex ontology design patterns can be asserted using different relational pattern definitions. Table 1 lists a translation of some relations that are used in biomedical ontologies to relational pattern definitions. Our approach can be restricted to unary or extended to arbitrary *n*-ary relations, *n *> 2. Unary patterns require a single variable symbol, while *n*-ary relational pattern definitions use the variable symbols ?X1, ?X2,...,?Xn. We implemented only binary pattern definitions to provide compatibility with the OBO Flatfile Format, and to enable graph-based access to OWL ontologies.

**Table 1:** Relational pattern definitions for selected relations

Relationship	OWLDEF Pattern
part-of	?X subClassOf part-of some ?Y
has-part	?X subClassOf has-part some ?Y
integral-part-of	(?X and not (part-of some ?Y)) or (?Y and not (has-part some ?X)) subClassOf Nothing
has-integral-part	(?X and not (has-part some ?Y)) or (?Y and not (part-of some ?X)) subClassOf Nothing
proper-part-of	?X subClassOf proper-part-of some ?Y
has-proper-part	?X subClassOf has-proper-part some ?Y
located-in	?X subClassOf located-in some ?Y
location-of	?X subClassOf location-of some ?Y
contained-in	?X subClassOf contained-in some ?Y
contains	?X subClassOf contains some ?Y
adjacent-to	?X subClassOf adjacent-to some ?Y
transformation-of	?X subClassOf transformation-of some ?Y
transformed-into	?X subClassOf transformed-into some ?Y
derives-from	?X subClassOf derives-from some ?Y
derived-into	?X subClassOf derived-into some ?Y
preceded-by	?X subClassOf preceded-by some ?Y
precedes	?X subClassOf precedes some ?Y
has-participant	?X subClassOf has-participant some ?Y
participates-in	?X subClassOf participates-in some ?Y
has-agent	?X subClassOf has-agent some ?Y
agent-in	?X subClassOf agent-in some ?Y
*realized-by*	?X subClassOf realized-by only ?Y
*realizes*	?X subClassOf realizes some ?Y
*lacks-part*	?X subClassOf not (has-part some ?Y)
*has-function*	?X subClassOf has-function some ?Y
*lacks-function*	?X subClassOf not (has-function some ?Y)
*has-function-realized-by*	?X subClassOf has-function some (realized-by only ?Y)

Emphasized relations are not a part of the OBO Relationship Ontology, but are included in its extensions.

### Extension to OBO syntax and semantics

We focus on applying relational pattern definitions to extend the OBO language and make the semantics of relations used in OBO ontologies explicit. For this purpose, we add an OWL definition of the relations used in an OBO ontology to the OBO language, and use this definition for the conversion to OWL [13]. A basic definition of an ontological category in the OBO Flatfile Format has the following form:

[Term]

id: term-id

name: term-name

is_a: term-id-super

relationship: relationship-id term-id-R

This definition states that a category with the identifier term-id and the label term-name stands in the is-a relation to the category with the identifier term-id-super, and in the relationship relationship-id to the category identified by term-id-R. The grammar of the currently used version of the OBO Flatfile Format lists several additional elements which a term definition may contain [1].

The key issue that the RO [7] attempted to resolve is the provision of a uniform and flexible interpretation of the relationship statements in the OBO Flatfile Format. Currently, translations to OWL for a relationship statement in the OBO Flatfile Format occurring in the definition of a category with the identifier term-id

relationship: relationship-id term-id-R

result in

term-id subClassOf: relationship-id some term-id-R

Such translations fix a particular interpretation of what a relation between two terms in the OBO Flatfiles designates. Although the intension of the OWL relation relationship-id is not specified, the relationship represented in the OBO flatfile, as a relationship between two terms (which represent categories), is defined uniformly using a new relationship between the instances of these categories; and this new relationship is used in an existential statement.

The typedef environment is used to define relationships in the OBO Flatfile Format, and assert basic axioms to these relations. We provide a minimal extension of the current OBO Flatfile Format to include OWL relational pattern definitions in the typedef stanza of the OBO flatfile. Since the OBO Flatfile Format is intended to be read both by machines and read by humans, we selected the Manchester OWL Syntax to represent the relational pattern definitions. We add an owldef statement to the typedef environment of the OBO Flatfile Format such that a relationship can be defined using a relational pattern definition:

owldef: owl-pattern-definition

Applying our method to extend the OBO Flatfile Format syntax and semantics permits a view on OBO ontologies where an ontology consists of (1) a list of relations that are used in the ontology, and (2) the actual domain content of the ontology. The actual domain content is intended to be modified and curated by domain experts. The list of relations and the definitions of the relations in this list can be developed and maintained in close collaboration by domain experts and experts in formal logics and ontology, similar to the approach taken in the RO. This leads to a reuse of existing resources and curation efforts, improve interoperability and the correct use of relations within domain ontologies.

### Implementation

We implemented the OBO Flatfile Format syntax and semantics [1] together with our extensions as a Java software. The software implements a generic parser for the OBO Flatfile Format. It represents the OBO ontologies as a list of ontological categories and a list of relation types. The relation types include the relational pattern definitions.

To convert this representation to OWL, we use the Manchester OWL API [14]. Every relation assertion that is not explicitly defined is translated using the standard semantics of the OBO Flatfile Format, i.e., as an existential statement. For example, if the relation **part-of **contains no owldef definition, its use in the definition of the class *Nucleus*

relationship: part-of Cell

will be translated as

Nucleus subClassOf (part-of some Cell)

On the other hand, when a relational pattern definition is available, the variable symbols ?X and ?Y are replaced with the first and second argument of the relation and then converted to an OWL axiom using an inline parser for the Manchester OWL Syntax of the Manchester OWL API. For example, the relationship statement

relationship: lacks-part Nucleus

in the definition of the class *Red blood cell*, together with the definition of **lacks-part**

owldef: ?X subClassOf (not has-part ?Y)

is first converted to the statement:

RedBloodCell subClassOf (not has-part Nucleus)

This statement is obtained by replacing the variable symbols with the names of the categories, *Red blood cell *and *Nucleus*. It is then parsed using the Manchester OWL API and added as an axiom to the OWL ontology.

Our implementation replaces the OBO parser that is available in the Manchester OWL API. Based on this implementation, we developed a command-line interface to convert ontologies from the OBO Flatfile Format to OWL. The source code is also available as a library so that our extension to the OBO Flatfile

Format can be integrated easily into software applications. Furthermore, we provide a web interface to perform conversions online to ease the adoption of our method. The web-interface can also be used to generate new OBO files that include OWL definitions. All software is freely available from our project website [15].

Further, we provide a prototype implementation to extract relational patterns from an OWL ontology. For this purpose, an OWL ontology is read using the Manchester OWL API. Based on a list of relational patterns and the list of all class names in the loaded OWL ontology, binary relations between classes are generated as OWL axioms: each class name in the signature of the OWL ontology is used to replace ?X in the pattern and then combined with all class names to replace ?Y in the same pattern. Consequently, all combinations of named classes are generated to fill variables in the relation patterns, leading to a list of OWL axioms.

Using the Hermit OWL reasoner [16], we then attempt to prove each of these OWL axioms and keep track of those that the reasoner could infer from the axioms asserted in the ontology. As a consequence, we obtain a list of theorems that hold in the ontology. We can convert these back to the OBO Flatfile Format by asserting the relations in the OBO ontology that were inferred using OWL reasoning.

The extraction of relational patterns from OWL ontologies is prototypically implemented using a naive approach. We currently use every pair of named classes and attempt to prove the axiom resulting from replacing ?X and ?Y in the definition patterns with the named classes. Consequently, this approach requires *n*^2 ^inferences using an OWL reasoner, where *n *is the number of named classes in the OWL ontology. Designing a more efficient algorithm is subject to future work.

## Results and Discussion

### Use cases

#### Missing parts or dispositions

The first obvious example where the current semantics fails is the class of **lacks **relations [9,10]. Using our extension, the relation **lacks-part **will be defined in the following typedef statement:

[Typedef]

id: lacks-part

owldef: ?X subClassOf: not has-part some ?Y

Then, a definition of the category *TaillessMouse is*

[Term]

id: TaillessMouse

name: Mouse that has no tail

is_a: Mouse

relationship: lacks-part Tail

Our mapping approach will yield the following OWL axiom for the OBO Flatfile statement:

TaillessMouse subClassOf: (not has-part some Tail)

The definition of the **lacks-part **relation can be refined by defining the **has-part **relation using the meta-property assertions in the OBO Flatfile Format. Using these meta-properties, **has-part **can be asserted to be transitive and symmetric. These assertions influence not only the interpretation of **has-part **but also of **lacks-part **when the definition we introduce is used. Another example where missing parts are relevant include red blood cells, which may be defined as cells which lack a nucleus as part. Similar to absent body parts, we can define absent dispositions, e.g., in the case of dysfunctional entities [17].

#### Functions and dispositions

However, **lacks **relations are not the only application of our OWL definition patterns. Many relations between categories do not imply the existence of an instance of one of the categories, but restrict the class membership *if there are instances*. They are used to assert that the instances of a class *C *stand in a relation *R only *to instances of a class *D *[18].

An example of such a relation is the **realized-by **relation between a disposition or function and a process that realizes the disposition or function [19,20]. Because some functions or dispositions are never realized, it would be false to assert that there is necessarily an instance of a process that realizes the function. Instead, *if *there exists a realization, it *must *be of a certain kind. Consequently, the definition of **realized-by **is

?X subClassOf realized-by only ?Y

In addition to the all-all pattern, more complex definitions can be used. For example, the relation **has-function-realized-by **[17] is a relation between a class of function bearers and a class of processes which are the realizations of functions borne by instances of the first class. We de ne **has-function-realized-by **as:

?X subClassOf: has-function some (realized-by only ?Y)

#### Implementing the OBO Relationship Ontology

In addition to domain-specific use cases, relational pattern definitions provide a means to implement and distribute the RO [7]. Relational pattern definitions make the definitions of relations between classes explicit and integrate them with OWL ontologies and biomedical ontologies using the OBO Flatfile Format. In contrast to definitions in first-order logic, an implementation in OWL takes advantage of the large number of tools and libraries that are available for OWL. In particular, an OWL implementation can be used to support automated reasoning with ontologies.

Our implementation of the RO was manually performed and ignores temporal arguments, because OWL does not support ternary relations. Once a standard OWL-based semantics for temporal arguments of RO relations is available, appropriate pattern definitions that include temporal arguments can be defined.

*Ternary *relational pattern definitions provide a means to avoid universal quantification over the temporal arguments in the RO relation. Except for the **derives-from **and **derives-into **relations, the implementation we provide is formally equivalent to the first order version of the RO without temporal arguments. In the RO, the relations **derives-from **and **derives-into **are defined using sequences of changes in identity, which we approximate by introducing a new relation between instances.

Table 1 shows the relational pattern definitions for the RO and some additional relations. The continued development of RO in the form of patterns would be beneficial, such that users and developers of ontologies do not merely use the same *name *of a relation, but the same definitions as well. A method of importing the RO into OBO ontologies would hide the logical complexity from ontology developers and users.

### Towards a standard semantics for the OBO Flatfile Format

Relational pattern definitions and using the OWLDEF method provide a flexible way to de ne relations using complex OWL statements in biomedical ontologies and the OBO Flatfile Format. However, owldef statements interfere with other parts of the OBO Flatfile Format. In particular disjointness, intersection and union statements do not inter-operate well with the OWLDEF method. The following definition of a category in the OBO Flatfile Format illustrates the problem:

[Term]

id: ID:1

intersection_of: ID:2

intersection_of: integral-part-of ID:3

The difficulty is that integral-part-of ID:3 is not a class description when the OWLDEF method is used. Instead, ID:1 integral-part-of ID:3 would translate into one OWL axiom. Axioms cannot be disjoint from classes (ID:2) in OWL.

However, the current translations of the OBO Flatfile Format to OWL do not provide an adequate semantics for this statement either, because the relation **integral-part-of **is not translated appropriately. One possible solution would be to disallow the use of relational statements in intersection, disjointness or union statements, and allow only class names as arguments. Another would be to restrict the kind of relation that can be used in these statements.

Solving this problem is subject to future research, and falls in line with the effort to provide a standard semantics for the OBO Flatfile Format that is compatible with currently available resources and allows expressive relation assertions between categories.

### Evaluation

To evaluate our method, we applied it to the Celltype Ontology (CL) [21]. We chose the CL due to its average size (1,062 classes), relative maturity and lack of formal definitions. The CL uses two relations, **is-a **and **develops-from**. The patterns for **is-a **and **develops-from **are ?X subClassOf: ?Y and ?X subClassOf: develops-from some ?Y. We implement the pattern for **develops-from **using the owldef statement in the OBO Flatfile Format:

[Typedef]

id: develops_from

name: develops_from

owldef: ?X subClassOf: develops-from some ?Y

The CL contains 1,253 **is-a **and 275 **develops-from **statements, i.e., 1,528 axioms that restrict CL categories using one of these two relations. We classify the generated OWL ontology using the Hermit OWL reasoner. Based on the classified OWL ontology, we attempt to prove the two patterns for each pair of named classes in the ontology. We use the Hermit reasoner to perform these inferences. Using this approach, we identify 9,497 **is-a **and 124,420 **develops-from **statements that we can add to the OBO Flatfile representation of the CL. The inferences are obtained from OWL reasoning on the semantics of the ontology, which take transitivity of **is-a **and **develops-from **relations into account.

The CL only uses the **is-a **and **develops-from **relations which are interpreted correctly by most OBO to OWL converters. Therefore, we used the Malaria Ontology [22] to evaluate our method. The Malaria Ontology uses the relation **realized-by **which should be defined as [18]:

[Typedef]

id: realized_by

name: realized_by

owldef: ?X subClassOf: realized-by only ?Y

Using our method, we infer 56 **realized-by **relations from three assertions of **realized-by **in the OBO version of the Malaria Ontology.

Furthermore, we added the axiom that *exon *is **integral-part-of **a *transcript *to the Sequence Ontology (SO) [23], in accordance with the SO developer's proposal for defining SO *classes *[24]. From this assertion, we could infer that *exon *stands in the **has-part **relation to *sequence feature and biological region*, neither of which was asserted in the OBO Flatfile implementation of the SO.

This shows that the application of our method provides a powerful means to complete the information in an ontology implemented using the OBO Flatfile Format through automated reasoning. In combination with our novel implementation of the RO, we provide a way to present the inferences of the assertions in a domain ontology to the curator so that they can be used to verify, correct and complete the ontology's content.

In our use-case using the Celltype Ontology [21], the conversion of OWL to OBO required 264 minutes on an AMD Opteron processor with 2.3 GHz and using 10 GB of memory. In the future, we will attempt to reuse already performed inferences and use heuristics to speed up the process of inferring the patterns.

### Future research

The main task for the future will be to work with the developers of the OBO Flatfile Format to agree on a common specification of the syntax and semantics of the OBO Flatfile Format. Several suggestions for developing the semantics of the OBO language were proposed [1,25], each with their own advantages and disadvantages. The closest to our approach is a proposed semantics [26] based on Common Logic [27]. However, Common Logic is, in general, undecidable and does not benefit from the variety of software tools and libraries that have been developed for OWL. We therefore suggest including our extension, or a variant of it, in a next version of the OBO Flatfile Format.

Furthermore, to use OWL reasoning for ontology engineering in the OBO Flatfile Format, we intend to include our extension in the OBO-Edit ontology editor. The current semantics of the OBO Flatfile Format, in particular the use of existential statements for relations, limits the possibilities for discovering inconsistencies in ontologies. Expressive owldef definition patterns that may include negation provide a means for automatic consistency verification and reasoning, and consequently support the ontology engineering process.

Performing the roundtrip between the OBO Flatfile Format and OWL would permit a seamless integration of OWL and graph-based languages such as the OBO Flat file Format. The current main limitation is the performance of extracting relational patterns from OWL using automated reasoning. To make our approach widely applicable within the Semantic Web community, more sophisticated algorithms must be investigated to extract relational pattern assertions between classes.

Although our main application is to provide a semantics for biomedical ontologies, OWL relational pattern definitions can also be applied to unstructured RDF data to provide a semantic layer and an interpretation of relations used in RDF stores. One use-case would be to apply our method to Linked Data [28]. Linked Data is a web of data where URIs denote things and links between URIs are expressed using RDF and represent relations between things. At least a fragment of the Linked Data cloud contains URIs of *classes*, not individuals, and relations between these classes are expressed in RDF. Similar to the OBO Flatfile Format, the semantics of the relation between classes is not made explicit. Using OWL relational pattern definitions, we can provide a way to represent some parts of the Linked Data cloud in OWL, utilizing the expressive semantics of OWL to formalize, structure and verify pieces of data. An implementation and evaluation of applying OWL relational pattern definitions to RDF and Linked Data is subject to future work.

## Conclusions

We developed a method to apply relational pattern definitions in biomedical ontologies, and we extended the syntax and semantics of the OBO Flatfile Format to allow the use of relational pattern definitions. The patterns we introduce are based on the assumption that any OWL class axiom in which variables *for classes *are used defines a relation between two classes. The patterns are formulated using an extension of the Manchester OWL Syntax, which is a human readable and writable syntax for OWL. The patterns can be used to provide a graph-based front-end for OWL ontologies.

To facilitate interoperability of our method with biomedical ontologies, we implemented an extension to the OBO Flatfile Format to include relational pattern definitions. Our proposal properly extends the OBO Flatfile Format to generate accurate and highly expressive OWL ontologies. Additionally, it permits the use of flexible interpretations of relations between classes.

We show the merit of our approach in several use cases. In particular, negation and universal quantification can be expressed in the OBO Flatfile Format when relational pattern definitions are used. Our approach is compatible with the OBO Relationship Ontology and permits an implementation of the OBO Relationship Ontology both in OWL and in the OBO Flatfile Format. For this purpose, we have translated the OBO Relationship Ontology to relational pattern definitions as required by our approach. Our proposal benefits the development of biomedical ontologies through the reuse of methods and software applicable in the Semantic Web. We suggest extending the OBO Flatfile Format by including relational pattern definitions in its syntax and semantics. Additionally, we propose a method for continued development of the OBO Relationship Ontology that makes the relationship definitions in the RO amenable for automated reasoning using OWL reasoners.

The application of our method does not only provide a means to make currently available biomedical ontologies amenable for automated reasoning using OWL software tools and libraries. The use of relational pattern definitions makes graph-based ontologies available in OWL, and makes complex OWL ontologies accessible in a graph-based form. Thereby, our method provides the means to gradually move the representation of biomedical ontologies towards a formal knowledge representation languages that incorporates an explicit semantics.

## Availability and requirements

• Project name: OBO2OWL-Patterns

• Project home page: http://bioonto.de/obo2owl

• Operating system(s): Platform independent

• Programming language: Java, Groovy

• Other requirements: Manchester OWL API 2.0 or higher

• License: Modified BSD License; the original BSD license was modified to remove the advertisement clause and thus make it compatible with free software licenses such as the GNU General Public License.

## Authors' contributions

RH conceived of the project and drafted the paper. HH and RH developed the semantics for the language extensions. RH and AO implemented the software. JK provided use-cases from biology, MD use-cases in the Semantic Web. HH, JK and DR supervised the project. All authors have read and approved the final version of the manuscript.
